# Impacts of livelihood assets on hydropower displacees’ livelihood strategies: Insights from the Tanahu hydropower project in Nepal

**DOI:** 10.1016/j.heliyon.2024.e34485

**Published:** 2024-07-11

**Authors:** Ribesh Khanal, Yuefang Duan, Thomas Stephen Ramsey, Sher Ali, Kaung Htet Oo

**Affiliations:** a College of Economics and Management, China Three Gorges University, 8 Da Xue Lu, Xiling District, Yichang, Hubei, 443002, China; b Research Center for Reservoir Resettlement, China Three Gorges University, 8 Da Xue Lu, Xiling District, Yichang, Hubei, 443002, China; c School of Environment, Northeast Normal University, 2555-Jingyue Road, Changchun, China

**Keywords:** Hydropower, Displacement, Livelihood, Sustainability, Nepal

## Abstract

Examination of livelihood assets is crucial for displaced people as it informs effective resource allocation and support. This study investigates the livelihood decisions of households displaced by Nepal's Tanahu Hydropower Project using multinomial logistic regression to evaluate how different assets affect their choices. Data were obtained using questionnaires, with a total of 185 questionnaires used for the analysis in this study. Descriptive and inferential statistics were used for data analysis. This study finds that physical assets substantially influence livelihood strategies, promoting diversification and nonfarming activities. Interestingly, social assets have a negative impact, whereas natural, financial, and human assets exert no significant effect. The study highlights the community's focus on economic stability, balancing immediate financial needs with long-term goals such as children's education. However, it reveals ongoing financial struggles, with an average daily income of only 1.962 USD and many households remaining in poverty, particularly those dependent on farming. The study also reflects on the mixed perceptions toward government policies, influenced by factors such as skill gaps and family aspirations. These findings provide essential insights for targeted support and resource allocation to displaced populations.

## 1 Introduction

Hydropower stands as the leading low-carbon electricity source worldwide and plays a pivotal role in the energy landscape, particularly in the Global South and emerging economies where dam construction is rapidly expanding [[1], [2], [3]]. Similarly, a recent study from the World Bank reported that approximately 733 million people lack electricity access, with many residing in the Global South [2]. Both national governments and private sectors promote hydroelectric projects as catalysts for economic growth, energy independence, and solutions to widespread energy deficits [[4], [5], [6]]. Hence, dams have been proposed as a viable solution for many developing and underdeveloped countries in the Global South [2].

However, dam projects are controversial. Despite their benefits, numerous studies have documented the detrimental effects of dams on local communities, their livelihoods, and their surrounding ecosystems, sometimes even failing to provide the anticipated energy access [[7], [8], [9], [10]]. Approximately 80 million people have been displaced globally because of dam projects [11,12]. Recent research indicates that new dams in regions such as Asia, Africa, and Latin America could diminish Gross Domestic Product (GDP), population, and land cover in adjacent areas [1].

Nepal, one such country in South Asia, holds a significant potential for hydropower, often compared with the oil wealth of Gulf countries. Although Nepal has the potential to generate up to 83,000 MW of power, only 43,000 MW is considered to be viable. However, they have only been able to produce 1129 MW so far [13,14]. In recent years, numerous hydropower projects have been established across the country, driven by the allure of clean energy generation, expanded electricity access, and revenue generation through export, hence raising the debate of hydropower displacement.

Literature shows that those who support hydropower, including dam authorities, governments, and engineering firms, argue that dams contribute to modernization, technological advancement, national development, and energy security, which they associate with improved health, education, and income levels [[15], [16], [17]]. Contrarily, those who oppose, such as activists, affected communities, and social justice organizations, criticize the socioecological and economic damage inflicted by these projects, highlighting the lack of participatory decision-making [[17], [18], [19]]. However, there are ways to compensate for losses and mitigate the risks faced by displaced people, including cash compensation, trading land for land, resettlement with compensation, and various forms of benefit-sharing [6,20,21]. Unfortunately, there is evidence that the lives of many displaced people have not been adequately restored due to the severity of impacts and the methodologies aimed at restoration and reconstruction in the post-resettlement period [8,20,22].

Despite the clear and substantial issues associated with displacement from large-scale development projects, there has been a surprising scarcity of research on involuntary relocation in Nepal. Before 2010, Bhattarai discussed displacement and resettlement laws and policies [23], and Rai focused on social inequality and power dynamics regarding cash compensation [24]. Similarly, Dixit et al. analyzed five preconstruction dam projects in Nepal using secondary data [25], providing only a broad overview of the potential impacts without deeply delving into the consequences or including firsthand accounts from locals at risk of displacement. More recent studies, such as that of Koirala, highlighted the need to understand the impacts of large hydropower projects in the early stages to effectively tackle displacement and resettlement issues [26]. Similarly, Khanal et al. emphasized the need to integrate environmental justice into large hydropower developments [27]. However, we did not find a study that analyzed the livelihood assets of hydropower displacees and their strategies in Nepal post-displacement.

Hence, to do so, this study employed the sustainable livelihood framework (SLF). This framework provides a valuable perspective for assessing the livelihood risk encountered by individuals displaced by hydropower projects [28]. This approach focuses on a household's ability to enhance its assets and capabilities over time in the presence of numerous stressors, such as environmental, economic, social, political, legal, and institutional variables. It has been recognized over time as an inclusive analytical tool and a practical means of identifying the variables contributing to the vulnerability of displaced communities and presenting varied solutions [29]. Moreover, it has been employed in several poverty alleviation and rural development programs worldwide [30].

In the context of sustainable livelihoods, this study delved into the “livelihood assets and objectives—livelihood strategies—livelihood outcomes” of individuals affected by hydropower projects. It aimed to explore strategies for reducing poverty among individuals involuntarily displaced by hydropower construction. Furthermore, it aimed to gain better understanding of the primary goals of these displacees, analyze their sentiments of optimism or pessimism toward resettlement policies, and identify which livelihood strategy proves most effective by measuring daily per capita income. The insights gained from this study will be important for policymakers to design targeted policies and programs for reducing poverty among hydropower displacees.

## 2 Literature review

Sustainable livelihood (SL) has substantially evolved since its introduction by Amartya Sen in 1983, initially aimed at addressing income poverty and later expanding to include developmental capabilities. The concept gained prominence with its inclusion in the 1992 United Nations Conference on Environment and Development, highlighting the importance of promoting stable livelihoods to eradicate poverty. Chambers and Conway provided a widely accepted definition, viewing livelihoods as a combination of capabilities, assets, and activities [31].

The concept of livelihood is differently understood in academic circles, and the viewpoints of research and analysis into SL vary. The sustainable livelihoods framework (SLF), developed by the UK's Department for International Development, became a cornerstone for analyzing livelihood challenges and strategies. In a recent scholarly study, the focus on SL has been directed toward four primary areas: poverty alleviation strategies [[32], [33], [34]], assessing livelihood risks among agricultural communities [35,36], exploring the accumulation of livelihood capital by farmers [37,38], and a broader examination of SL across different contexts [39,40]. A study by Young and Jacobsen on individuals relocating to urban areas within conflict zones highlighted substantial disruptions in basic living conditions, including access to food and stable housing [41]. Fred investigated the living conditions of rural populations in southern Africa, focusing on environmental factors, educational levels, and the economic disparity caused by fluctuating prices affecting farmers' livelihoods [42]. Cernea explored the detrimental impact of land loss on farmers, indicating that such displacement often leads to a new form of poverty, rendering their livelihoods unsustainable [43]. This is consistent with Cernea's main risk assessment of forced displacement and the early stage of the process of impoverishment [44]. Additional research pointed to environmental disasters and market volatility as critical factors impacting agricultural livelihoods, with affected communities often struggling to effectively respond to these external challenges [45]. A study by Kennedy on Kenya's environmental management highlighted the importance of adopting appropriate living strategies to ensure sustainability for resettlers [46]. Moreover, Mhongera and Lombard advocated for increased government intervention to support underprivileged groups, such as young girls, who are disproportionately impacted by insufficient public services and funding, emphasizing the need for comprehensive support systems to foster SLs [47].

A study on the resettlement associated with hydropower projects has largely concentrated on the significant challenges these projects pose to both economic and social sustainability in the reservoir areas. Studies conducted by Downing, Singh and Hiremath, Zhang et al., and Babu and Datta have detailed how these relocations impact the surrounding environment [[48], [49], [50], [51]], with significant ecological consequences [[52], [53], [54]]. Jing highlighted the need for resettlement strategies that not just minimize psychological trauma but also enhance the quality of life as well as the necessity to effectively tailor these strategies to improve both the employment prospects and overall livelihoods of immigrants [55]. In a related study, Reddy et al. used a sample from India to analyze the effects of organic agriculture on farming at a microlevel and its broader social implications using economic models such as the double-difference method and economic surplus model [56]. This research suggests that targeted organic farming strategies could substantially enhance food security and economic outcomes for farmers if the government strategically promotes organic cultivation, certification, and marketing based on regional needs. In addition, Reddy examined how families displaced by the Tehri Hydropower Project adapted to urban environments, focusing on their needs for job skills and market opportunities [57]. This study emphasized the importance of local resource utilization and the expansion of employment training programs to help resettled families improve their living conditions and integrate into new communities.

The concept of vulnerability initially applied to studies on natural disasters has evolved to encompass a broader range of issues, including financial instability, disaster susceptibility, climate change effects, resource depletion, and ecosystem fragility. This expansion reflects the multifaceted nature of vulnerability, which Gallardo and Koomson et al. described as a collection of interrelated natural and man-made phenomena that pose threats to the livelihoods of individuals and households [58,59]. McCulloch and Calandrino defined vulnerability in terms of economic risk, particularly as the likelihood of falling below an accepted monetary threshold that categorizes individuals or households as impoverished [60]. This perspective highlights that vulnerability is not one-dimensional but involves various risks and uncertainties that affect livelihoods. For example, natural events or human actions can disrupt the socioeconomic well-being of communities, leading to significant challenges and adverse consequences in livelihoods [61,62]. Hence, vulnerability can arise from environmental forces [63,64] or the decisions and actions within a household [65,66].

Research into the vulnerability of livelihoods has three main goals. First, it assesses vulnerabilities by examining environmental conditions, the availability of livelihood capital, and policy impacts [[67], [68], [69]]. Second, it enhances resilience by measuring poverty risks and developing early-warning systems [70,71]. Third, it develops methods to evaluate vulnerability, such as creating indices and using data envelopment analysis to quantify impacts [72]. These studies emphasize the critical association between livelihood capital, capability, and rights, highlighting the importance of effective governance in enhancing community resilience and reducing poverty [42].

Similarly, several studies on resettler'' livelihood were conducted globally. Most of the recent research on reservoir projects, particularly in the context of SLs, has come from China due to its ongoing hydroelectric projects. A current research by Chinese scholars explores the vulnerability of these communities, examining various factors influencing their livelihoods, the risks they face, and the strategies they employ to cope with displacement [42,[73], [74], [75]]. These researches indicate that various factors affect the livelihoods of resettlers, with the ecological environment substantially influencing economic and social development and ultimately quality of life. Consequently, environmental indicators are crucial in assessing SLs [42]. Research has also demonstrated the importance of social networks and risk identification for the SLs of rural resettlers [76]. Other factors influencing resettlers’ survival and sustainability include policy guarantees, livelihood security, and economic foundations [77].

Outside China, Ariyani et al. examined the hardships faced by those displaced by Indonesia's Saguling Dam and found significant challenges in livelihood reestablishment due to lost occupations, resources, and inadequate cash compensation [78]. The study also highlighted the pivotal role of sociographic localities in determining social capital, which is essential for livelihood reconstruction. In a different context, Phonepraseuth analyzed the Nam Theun 2 Hydropower Project in Laos and demonstrated that careful planning and livelihood development initiatives can mitigate resettlement risks [79]. This project significantly enhanced access to various livelihood assets, leading to improved living conditions that extended beyond economic gains. Similarly, Kwadwo Owusu et al. explored the impact of livelihood support programs after the construction of Ghana's Bui Dam [80]. Their findings suggest that interventions such as cage aquaculture, weaving, and pottery have positively influenced socioeconomic activities in the resettled communities. However, challenges such as poor soil fertility, inadequate fishing equipment, and delays in land compensation have hindered broader revitalization efforts.

Research on SLs in Nepal has mainly focused on rural poverty reduction. A study by Khatiwada et al. showed that livelihood diversification and factors such as education, access to credit, and infrastructure are key to adopting better strategies [81]. Pandit et al. found that agroforestry systems improve livelihoods through economic benefits and conservation [82]. A DFID study (NPC/DFID, 2013) reported that the Koshi Hills region has seen significant improvement in quality of life and poverty reduction since the 1970s, largely driven by the efforts of the people themselves, with development interventions playing a supporting role [83]. Zhang and Fang assessed vulnerability in the Koshi River basin using the SLF, highlighting multiple climate-induced hazards impacting livelihood capitals [84]. Giri investigated the relationship between livelihood capital and strategies among the Dalit community and found a lack of opportunity and adoption of unsecured strategies due to societal constraints [85]. However, we did not find any research that employed the SLF to understand the livelihood situation of displaced people in Nepal.

Although extensive research on resettlement exists globally, there is limited research using the DFID model, specifically for hydropower displacees outside China. This is crucial as the literature suggests that displacement often results in “new poverty” due to the loss of natural resources, joblessness, financial deterioration, and marginalization. Institutional processes and organizational structures influence access to assets and shape livelihood activities, whereas vulnerability affects access to resources and livelihood options. Therefore, quantifying the sustainability of livelihoods, identifying weaknesses, and providing accurate policy support are crucial for resettlers and for achieving comprehensive rural development in resettlement areas. This study aimed to provide a foundation for effectively addressing the livelihood problems of resettlers and fostering their steady development.

However, some limitations exist within the SLF, particularly in practical application. It requires significant time, financial, and human resources, which development projects often lack. In addition, the emphasis on a holistic understanding of assets can lead to information overload, potentially obscuring the core issue of income poverty. Furthermore, improving the livelihoods of one group may negatively impact others, creating ethical dilemmas in prioritizing interventions. Despite these challenges, the widespread adoption of SLF justifies its use as our analytical framework for this study.

## 3 Methodology

### 3.1 Analytical framework

This study employed the SLF as the analytical framework to specifically address the dynamic interactions between livelihood assets, strategies, and outcomes within the context of communities displaced by dam construction in Nepal. The rural setting of Nepal, where many dwellers rely on land for subsistence agriculture amidst a rapidly growing population and a deteriorating natural environment, significantly influences the type of livelihood assets available [86,87]. In these communities, the livelihood assets typically include human, physical, financial, social, and natural capitals, which are foundational for developing various livelihood strategies that these displaced individuals and households may adopt.

Natural assets include natural resources such as farmland, which is crucial for farmers in impoverished areas to sustain their livelihoods and reduce vulnerability [32,52]. Financial assets comprise savings and regular income streams, including remittances and government transfers such as pensions and labor income, providing financial stability [52,88]. Both natural and financial assets are important for household production and resource management. Social assets include the networks, norms, and trust that facilitate cooperative actions [42,89]. These social networks are vital for displacees to access resources and meet social and economic needs. Meanwhile, human assets refer to the collection of skills and talents that enable individuals to pursue different livelihood strategies and achieve their goals [69]. Investing in human capital through education and training helps farmers transition to nonagricultural sectors, promoting SL diversification [52,88]. Physical assets, including infrastructure (roads, transport, buildings, water supply, energy, communications) and production tools and technology, are crucial for societal development [88]. These assets enable the production of essential commodities, and their absence significantly contributes to poverty because of inadequate services and infrastructure [2,69].

When classifying livelihood strategies, various approaches can be adopted, including the asset-based, activity choice, and income-based approaches. The asset-based approach classifies livelihood strategies from the perspective of input, focusing on asset allocation across different activities or asset portfolios [90]. However, this approach struggles to capture nonproductive income-generating activities that do not easily involve measurable asset inputs, such as investment, retirement, or transfer payments [91]. Meanwhile, the income-based approach classifies livelihood strategies from the perspective of output [92] based on income from specific sources such as nonfarm, forest, or cash transfer incomes. Despite its utility, the income-based approach has inherent drawbacks, including considerable variations due to the stochastic nature of income and the inability to distinguish asset and activity differences among households within the same income-based group [91].

Compared with these, the activity choice approach, as proposed by Nielsen et al., classifies livelihood strategies from the perspective of the process [93]. It emphasizes the connection between livelihood assets and outcomes through a sequence of income-generating activities, thereby associating the stock concept of assets to the ex-post flow of income typically used to measure livelihood outcomes.

Considering the traditional economic activities in certain regions, which heavily rely on locally available natural resources such as agriculture, livestock, and forestry, the livelihood strategies adopted by these communities often reflect a deep dependence on these resources. This framework categorizes strategies into three primary types: farming strategy, nonfarming strategy, and mixed strategy. Each strategy highlights the diverse ways in which communities strive to rebuild and sustain their livelihoods, adapting to the loss of natural assets and the opportunities or constraints presented by their new settings. For instance, those with access to some remaining or new agricultural land may continue with farming strategies, whereas others may switch to nonfarming strategies, such as small-scale businesses or wage labor, if their natural assets are severely diminished. The mixed strategy involves a combination of both, often seen in households attempting to diversify their income sources to enhance resilience against future shocks.

**Fig. 1:**
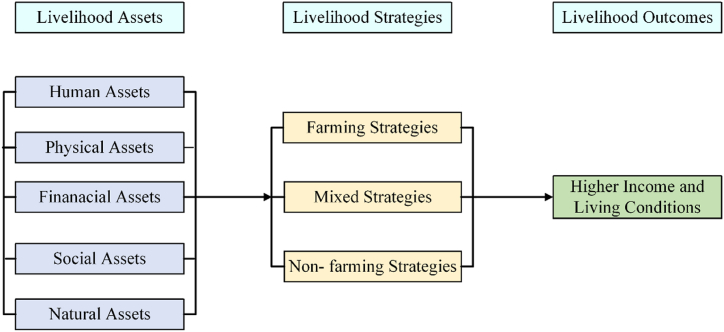
Analytical framework of the research.

Finally, the fifth component of the SLF is livelihood outcomes. It refers to the concrete results achieved through various livelihood strategies. These outcomes can include increased income, improved well-being, reduced vulnerability, enhanced food security, and more sustainable resource use. The outcome of these strategies is measured by “higher income,” as suggested by Khatiwada et al. [81], and by the subjective evaluation of the respondents of their living conditions, as suggested by Shen [94]. These metrics serve as a crucial indicator of success and sustainability. Fig. 1 presents the analytical framework of our research.

### 3.2 Project description

**Fig. 2:**
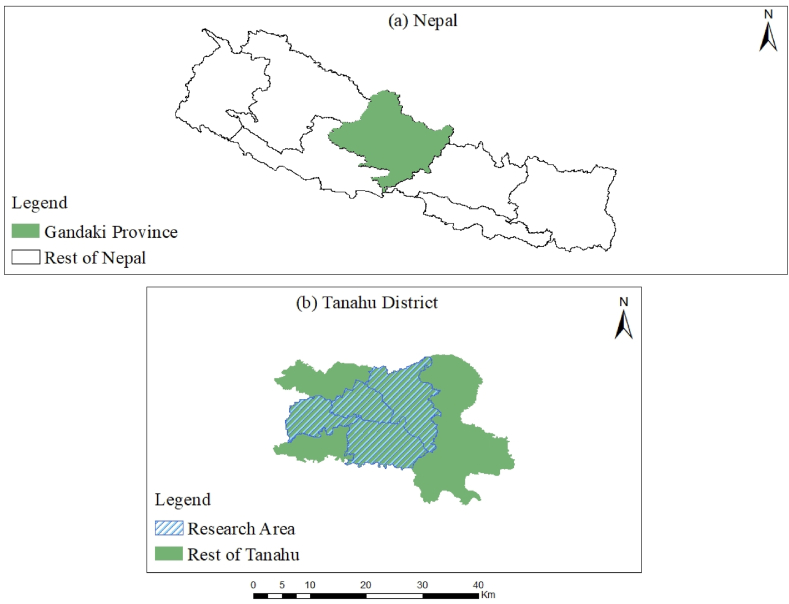
Study area map (a) Map of Nepal showing the Gandaki Province. (b) Map of Tanahu District highlighting the research area.

In 2012, the Nepal Electricity Authority established a fully owned subsidiary named Tanahu Hydropower Limited, which has a capacity of 140 MW. This storage hydroelectric project is located approximately 150 km west of Kathmandu in the Tanahu District of Nepal's Gandaki Province, near the city of Damauli on the Seti River. The project has received a $505 million funding from the Asian Development Bank (ADB), Japan International Cooperation Agency, European Investment Bank, and Government of Nepal. The objective of the Tanahu Hydropower Project is to provide a stable supply of electric power from renewable sources, catering to the growing demand within Nepal's interconnected power network. To achieve this objective, the project not only bolsters the country's economy and enhances the quality of life for its citizens but also facilitates the mitigation of the impacts of climate change. The project falls under Category A, which includes projects for involuntary resettlement and indigenous people, according to the 2009 ADB Safeguards Policy Statement (SPS). To adhere to the SPS guidelines, the Project Preparation Technical Assistance Consultant updated the Resettlement and Indigenous Peoples Plan for the Hydropower Component in October 2018. The implementation of this project has affected a total of 560 households and resulted in the permanent acquisition of 72.3 ha of privately owned land. Furthermore, various public resources including water supplies, temples, cemeteries, resting spots, suspension bridges, and footpaths, have been affected. Within the sample population, 72 % of the homes, amounting to 396 households, have been identified as vulnerable due to the project's ramifications. Fig. 2 shows the map of the research area.

### 3.3 Livelihood restoration and rehabilitation plan of the Tanahu hydropower project

The project area's livelihood is predominantly dependent on agriculture, with minimal reliance on other income sources, such as trade, business, remittance, government jobs, wages from labor, and pensions. The water from the Seti River is not used for irrigation in the project area, and only a few households are mainly dependent on fishing, primarily engaging in recreational fishing. Thus, the primary impact is expected to be on farmers. The livelihood restoration plan encompasses both land- and nonland-based programs. Land-based programs, such as replacement land provision, are not feasible due to land unavailability. The focus is instead on the enhancement of land productivity for those who lose a portion of their cultivated land. Nonland-based strategies include compensation, skill training, project-related employment, direct credit for small businesses, and support for income-generating activities.

In the short term, the plan aims to provide immediate assistance to affected households, which includes full compensation for land and structures before relocation, financial and life skill training, relocation subsistence allowances, subsidized inputs for agriculture in the initial years, restoration of leased land to titleholders, temporary employment opportunities in construction activities, and special assistance for vulnerable individuals and households. The plan seeks to sustain income sources beyond the implementation period of the project for the long term. The plan involves employment programs, microcredit facilitation, establishment of linkages to district-level assistance programs, and continuation of support under the community development strategy (CDS) beyond project implementation. Furthermore, the plan integrates with the broader CDS during the construction phase.

Income restoration assistance will be extended to 200 households severely impacted by the project, with 150 (75 %) of these households being identified as indigenous and marginalized communities (IP households). These vulnerable households will receive a range of support measures aimed at revitalizing their livelihoods. This support includes a 90-day cash aid equivalent to the local agricultural wage rate, a special assistance grants of NR 10,000 allocated to each affected vulnerable household to expedite their livelihood restoration, and the opportunity for at least one member from each affected household to participate in vocational training and skill enhancement programs, tailored to their individual preferences and needs. To further alleviate the economic strain, the project contractors will prioritize offering temporary employment opportunities at the construction sites to the affected persons (APs). APs residing below the poverty line will be prioritized, ensuring equitable access to these job opportunities. Moreover, the plan addresses gender equality and social inclusion through events and discussions that actively involve women in decision-making processes and training preferences. This inclusive approach strives to guarantee that every member of the affected communities reaps the benefits of the livelihood restoration efforts (Tanahu Hydropower Project: Combined Resettlement and Indigenous Peoples Plan, 2018).

### 3.4 Data collection and questionnaire

For this study, data collection was conducted among individuals displaced by the Tanahu Hydropower Project in Nepal using questionnaires, with the fieldwork spanning from October 2022 to February 2023 across the rural municipalities of Rising, Myagde, Vyas, and Bhimad. To ensure a diverse and representative sample, we employed a stratified sampling technique. With the assistance of a social mobilizer, we categorized the displaced population into distinct subgroups based on specific characteristics reflective of the four resettled locations. We initially collected about 40 preliminary samples to refine the questionnaire and better capture key socioeconomic characteristics. Table 1 presents the livelihood asset indicators used and their corresponding literature sources.

**Table 1:** Livelihood asset indicators and literature.

Assets	Indicators	Description	Literature
Human Capital	Attainment of household labor education	0) illiterate; 3) semiliterate; 6) primary school; 10) secondary Level; 12) high school or technical school; 16) undergraduate; 18) graduate or above	[52,88,98]
	% of migrant workers	% of migrant workers of the total family members	[52,88]
	Types of insurance household has bought	1) no insurance; 2) term life insurance; 3) whole life insurance; 4) money back policy; 5) endowment policy	[52,88]
Physical Capital	Number of agricultural tools	Number of agriculture equipment and vehicles	[52,88]
	Number of household assets	bicycle, motorcycle, car, TV, refrigerator, washing machine, mobile phones, computer, and fan or air-conditioner	[52,88]
Financial Capital	Household savings	Current household savings amount (NPR)	[33,81,99]
	Household loan	Current household loan amount (NPR)	[81,98,100]
Natural Capital	Area of agriculture Land	Actual land holding in ropani	[52,81,94,100]
Social Capital	Degree of immigration policy perfection	1) no perfection at all; 2) no perfection; 3) slight perfection; 4) some perfection; 5) great perfection	[52]
	Difficulty in democratic decision making	1) very difficult; 2) difficult; 3) fair; 4) easy; 5) very easy	[52]
	Contact with relatives abroad	1) never; 2) seldom; 3) sometimes; 4) often; 5) almost always	[52]

Note: NPR stands for Nepalese Rupees; ropani is the local land measurement system (1 ropani = 0.05087 he).

Before the questionnaire distribution, a consent form was read aloud to respondents. They were informed about the purpose of the study and their rights, and verbal consent was obtained from each participant to ensure ethical compliance and understanding. This process was crucial for maintaining the integrity and ethical standards of our research.

The final stage of data collection involved random distribution of questionnaires to members of the displaced communities. Each interview took approximately 20–30 min, with some respondents requiring the questionnaire to be read to them verbally to ensure clarity and comprehension. When determining the optimal sample size for a study, several factors need to be considered, including the variability within the population, the desired accuracy level, and the intended statistical analysis of the results. In the case of our study on the total population of 560 hydropower displacees, different scholarly opinions suggest varying sample sizes.

Krjcie and Morgan recommended a sample size of 234 for populations approximating 600 [95]. Similarly, Nardi and Suskie recommended a minimum sample size of 30 % for populations smaller than 1000 to achieve adequate representativeness [96,97]. For our population of 560, this would imply a sample size of at least 168 individuals. Our selected sample size of 180, which constitutes 33 % of the hydropower displacees, closely aligns with these recommendations.

### 3.5 Empirical modeling

In the analysis of livelihood assets and strategies, both multinomial logit (MNL) and multinomial probit (MNP) models are frequently used. However, when evaluating the practical advantages of MNL over MNP, several factors come to the forefront. MNL models are particularly valued for their computational simplicity and efficiency and are crucial when managing a large array of choices, where the need of MNP for complex integrations could lead to computational difficulties [101]. The stability and robustness of MNL make it particularly effective for studies with limited sample sizes or less complex choice scenarios [102]. In addition, the ease of interpreting the odds ratio coefficients of MNL facilitates clear communication of results [103]. Empirical comparisons often reveal minimal differences in the predictive accuracy between the two models, indicating that the simpler computational approach of MNL does not sacrifice performance [101,102]. Similarly, research with a small sample size has used multinomial logistic regression in livelihood studies [104]. Hence, we applied this method in our study.

To analyze the impact of livelihood assets on farmers’ preferred livelihood strategies, we employed multinomial logistic regression. This approach is suitable for our categorical dependent variable, representing three common livelihood strategies: farming, mixed livelihoods, and nonagricultural activities, with assigned values of 0, 1, and 2, respectively. We aimed to determine the likelihood (P) of farmers selecting specific livelihood strategies based on a set of independent variables (livelihood assets). Equation (1) shows the formula used to measure the relationship between livelihood assets and strategies:

(1) $$P\left(y_{i}=j\right)=\frac{e^{x_{i\beta_{j}}}}{{\sum^{j}j=0}^{x_{i\beta_{j}}}}$$

Here, “i” denotes the sample; “j,” the chosen livelihood strategy; “X_i_,” the livelihood assets; and “β_j_,” the estimated parameter. “Farming” was set as the reference strategy (Y_0_), whereas “mixed” and “nonfarming” were assigned values Y_1_ and Y_2_ [29]. To fulfill the assumptions of logistic regression, such as independence and noncollinearity, we conducted principal component analysis (PCA) on the quantitative socioeconomic data related to displacees. Statistical Package for the Social Sciences (SPSS) 25 was used for data analysis.

## 4 Results

### 4.1 Description of the samples

#### 4.1.1 Demographic profile of the respondents

Table 2 presents the demographic profile of the surveyed respondents. The survey sample consisted of 185 respondents, including 135 men and 50 women. The age distribution was spread across various age groups, with 34 respondents aged 18–30 years, 48 respondents aged 31–40 years, 53 respondents aged 41–50 years, and 50 respondents aged over 50 years. Their educational levels varied, with 36 respondents being illiterate, 65 having primary education, 55 having secondary education, and 29 possessing higher education or a diploma. The family sizes were 107 households consisting of 4 or less members, 32 having 5 members, 37 having 6 members, and 29 having more than 7 members.

**Table 2:** Demographic profile of the respondents.

Gender	Male	Female		
Age	135	50		
	18–30	31–40	41–50	Over 50
	34	48	53	50
Educational levels	Illiterate	Primary education	Secondary education	Higher education or diploma
	36	55	65	29
Family size	4 or less members	5–6 members	7–8 members	More than 8 members
	86	67	17	15

#### 4.1.2 Expectations and evaluations of compensation policies

##### 4.1.2.1 Land expropriation compensation

During our survey, we found that all the respondents opted for cash compensation, which was completed in 2012. The land expropriated ranged from 0.009 ha (3 ana) to 0.915 ha (288 ana). Similarly, the cash compensation ranged from Rs. 3,00,000 Nepali rupees (USD 2280) to 60,000,000 (USD 4,56,000). (Note: The exchange rate was based on April 26, 2023, 1 NPR = 0.007639 USD and 1 ana = 0.0031 ha) All the 185 residents we interviewed had lost their agricultural land, thus becoming economically displaced.

##### 4.1.2.2 Policy satisfaction and reasons

**Fig. 3:**
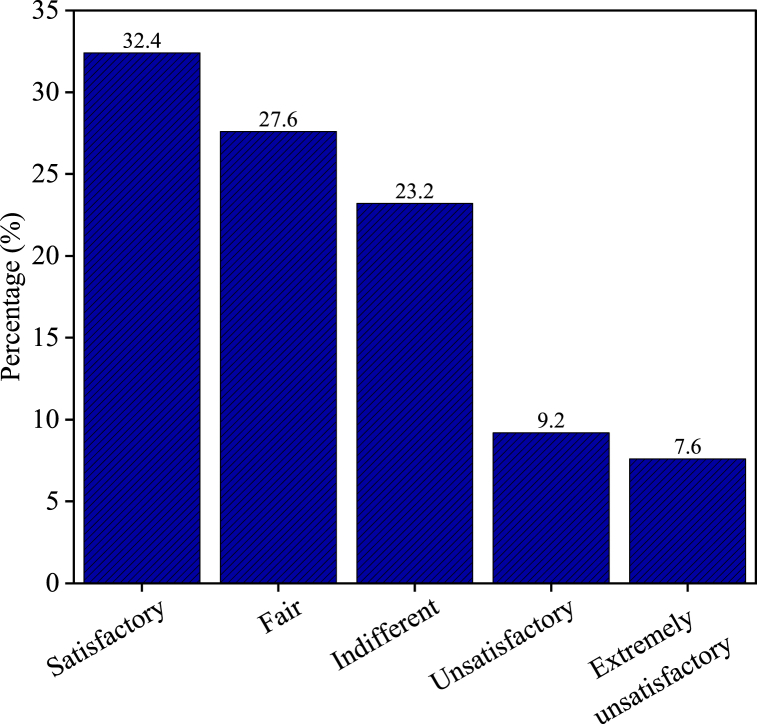
Level of compensation satisfaction.

Fig. 3 presents the levels of compensation satisfaction among the respondents displaced by the Tanahu Hydropower Project. The findings showed that 60 (32.4 %) respondents were satisfied with the compensation they received. A slightly smaller group, comprising 51 (27.6 %) respondents, considered the compensation to be fair. A total of 43 (23.2 %) respondents were indifferent about the compensation. Meanwhile, 17 (9.2 %) respondents were unsatisfied and 14 (7.6 %) were extremely unsatisfied with the compensation provided.

Similarly, Table 3 outlines the reasons for the compensation satisfaction among respondents displaced by the Tanahu Hydropower Project. A total of 126 (68.1 %) respondents attributed their satisfaction to reasonable compensation policies. In addition, 19 (10.3 %) respondents reported improved living conditions and infrastructure facilities as a reason for their satisfaction. An equal number of respondents (14 individuals [7.6 %]) cited increased income and more employment opportunities as reasons for their satisfaction. Furthermore, 11 (5.9 %) respondents felt that their living standards have improved due to the compensation. Only 1 (0.5 %) respondent mentioned enjoying urban welfare with an urban household as a reason for their satisfaction.

**Table 3:** Reasons for compensation satisfaction.

Items	Frequency	Percentage
Reasonable compensation policies	126	68.1
Improved living conditions and infrastructure facilities	19	10.3
More income	14	7.6
More employment opportunities	14	7.6
Higher living standard	11	5.9
Enjoying urban welfare with urban household	1	0.5
Total	185	100

Table 4 presents the reasons for compensation dissatisfaction among respondents displaced by the Tanahu Hydropower Project. The most significant reason, as reported by 52 (28.1 %) respondents, was reluctance to abandonment of their land. A substantial portion (33 respondents [17.8 %]) cited higher living costs and increased daily expenditures as sources of dissatisfaction. A total of 21 respondents (11.4 %) felt that the compensation fees for land expropriation were higher in other places, leading to dissatisfaction. In addition, 20 respondents (10.8 %) were concerned about an unsecured future life without a stable income after losing their land and 18 (9.7 %) were dissatisfied due to unemployment following the loss of land.

**Table 4:** Reasons for compensation dissatisfaction.

Items	Frequency	Percent
Reluctance to land abandonment	52	28.1
Higher living cost and increased daily expenditure	33	17.8
Higher compensation fees for land expropriation in other places	21	11.4
After land lost, without stable income, unsecured future life	20	10.8
Without land, unemployed	18	9.7
Compensation fees for land expropriation much lower than the land value	14	7.6
Land expropriation procedures not open and compensation not transparent	8	4.3
Higher compensation fees obtained by households for later land expropriation	7	3.8
Greater income gap between families	4	2.2
Housing relocation and resettlement policies to be improved	4	2.2
Compensation too low to keep living standard before land lost, dropped living quality	2	1.1
Social security policies to be improved	2	1.1
Total	185	100

Other reasons for dissatisfaction include compensation fees being much lower than the actual land value (14 respondents, 7.6 %) and the perception that land expropriation procedures are not open, and compensation is not transparent (8 respondents, 4.3 %). Seven respondents (3.8 %) reported that compensation fees obtained by households for later land expropriation were higher, creating dissatisfaction. Income disparity between families was a concern for 4 respondents (2.2 %), and the same number (4 respondents, 2.2 %) believed that housing relocation and resettlement policies need improvement. Lastly, 2 respondents (1.1 %) felt that compensation was too low to maintain their previous living standard, and another 2 respondents (1.1 %) thought that social security policies required improvement.

#### 4.1.3 Livelihood conditions of displacees

##### 4.1.3.1 Livelihood assets

As mentioned in the analytical framework, we used five livelihood capitals for the econometric analysis of this study. Table 5 presents descriptive statistical information on the livelihood asset situation among the displaced households. For human capital, the average level of household labor education is 7.3 years. The percentage of migrant workers among family members averages 49.8 %, with some families having no migrant workers and others having up to 100 %. As regards insurance, households have an average insurance type score of 3.4 on a scale from 1 (no insurance) to 5 (endowment policy).

**Table 5:** Descriptive statistics livelihood assets (independent variables).

Assets	Indicators	N	Minimum	Maximum	Mean	Std. Dev
Human	Labor force education	185	0	16	7.3	5.1
	Migrant labor %	185	0	100	49.8	21.8
	Insurance	185	1	5	3.4	1
Physical	Agriculture tool	185	0	9	4.4	2.4
	Household assets	185	5	20	13.1	3.7
Financial	Household savings	185	0	920000	76275.7	99502.8
	Household loans	185	0	25000000	774189.2	3170534
Social	Degree of immigration policy perfection	185	1	5	3.2	1.3
	Difficulty in democratic decision-making	185	1	5	3.3	1.1
	Contact with relatives abroad	185	1	5	3.6	1.1
Natural	Land area	185	1	35	8.9	7.7

(Note: Ropani is the local land measurement system [1 ropani = 0.05087 ha]. The exchange rate was based on April 26, 2023.).

In terms of physical capital, households own an average of 4.4 agricultural tools. The mean number of household assets, including items such as bicycles, motorcycles, cars, TVs, refrigerators, washing machines, mobile phones, computers, and fans or air-conditioners, is 13.1. Financial capital statistics revealed that the average current household saving amount is NPR 76,275.7. The average household loan is NPR 774,189.2. Similarly, for natural capital, the average agricultural land area is 8.9 ropani. Lastly, social capital indicators indicate that the mean degree of immigration policy perfection is 3.2. The difficulty in democratic decision-making averages 3.3. The frequency of contact with relatives abroad averages 3.6.

##### 4.1.3.2 Displacees’ opinion on livelihood goals

To develop resilience and foster long-term recovery among displaced individuals, it is important to understand their livelihood objectives and capabilities. Our questionnaire focuses on three key aspects: future life expectations, reasons for optimism, and reasons for pessimism. Respondents can choose from five future life expectation options, which include income improvement, livelihood security enhancement, and social status elevation. The questions regarding optimism and pessimism reasons reflect their confidence in achieving livelihood goals and the underlying factors driving their attitudes, providing valuable insights into resilience-building efforts.

**Fig. 4:**
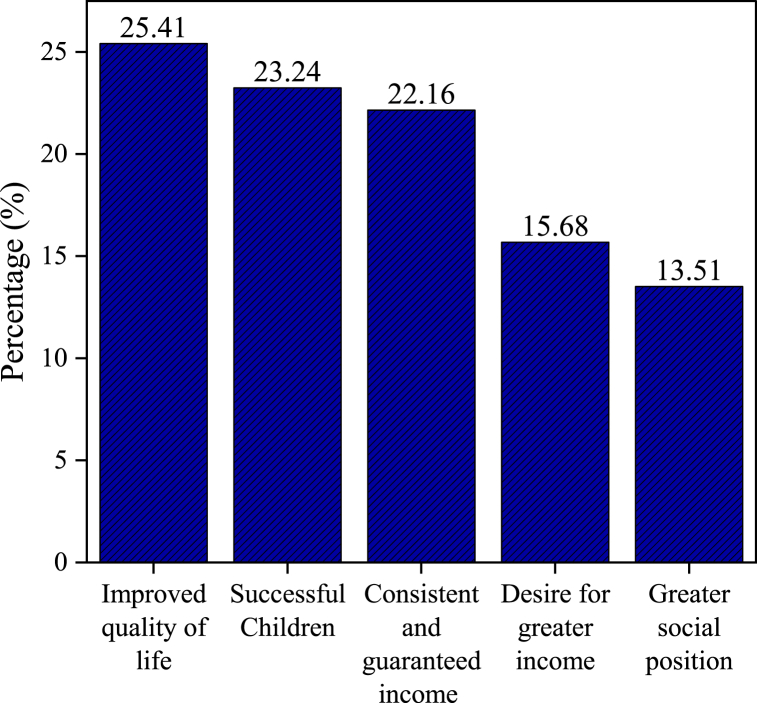
Future life expectation.

In the context of the surveyed population, livelihood goals are measured through their expectations for future life, as presented in Fig. 4, encompassing a range of aspirations. Predominantly, 47 respondents (25.41 %) expressed a primary desire for improved life quality, highlighting their pursuit of enhanced living conditions. Moreover, 43 individuals (23.24 %) prioritized the success of their children as a significant goal. Financial security is a crucial objective, with 41 respondents (22.16 %) seeking a consistent and guaranteed income. Furthermore, 29 participants (15.68 %) aimed to achieve greater personal income, reflecting a broader economic ambition, whereas 25 respondents (13.51 %) focused on elevating their social position within their community.

We asked respondents about their confidence in accomplishing their livelihood objectives and the reasons for their optimism regarding future livelihood strategies based on their opinions about livelihood goals. Table 6 presents the grounds for optimism and pessimism among those affected by the hydropower resettlement policy. Notably, respondents' optimism is substantially driven by the belief in favorable policies 54 (29.19 %), the anticipation of children's success 43 (23.24 %), the prospect of development opportunities 33 (17.84 %), a strong work ethic 30 (16.22 %), and trust in government support during the times of need 25 (13.51 %). Conversely, pessimism is predominantly fueled by concerns over insufficient government support 77 (41.62 %), the burden of family responsibilities 51 (27.57 %), insufficient technical skills 35 (18.92 %), and low literacy levels 22 (11.89 %). These findings indicate the importance of policy implementation and support systems in influencing persons' outlooks in the context of hydropower resettlement.

**Table 6:** Reasons for optimism and pessimism.

Optimism	Favorable policies	Successful Children	Development opportunities	More personal hard work	Government support in time of need
Percentage	29.19	23.24	17.84	16.22	13.51
Pessimism	Insufficient government support	Family burdens	Inadequate technical skills	Low literacy level	
Percentage	41.62	27.57	18.92	11.89	

##### 4.1.3.3 Displacees’ livelihood strategies

**Fig. 5:**
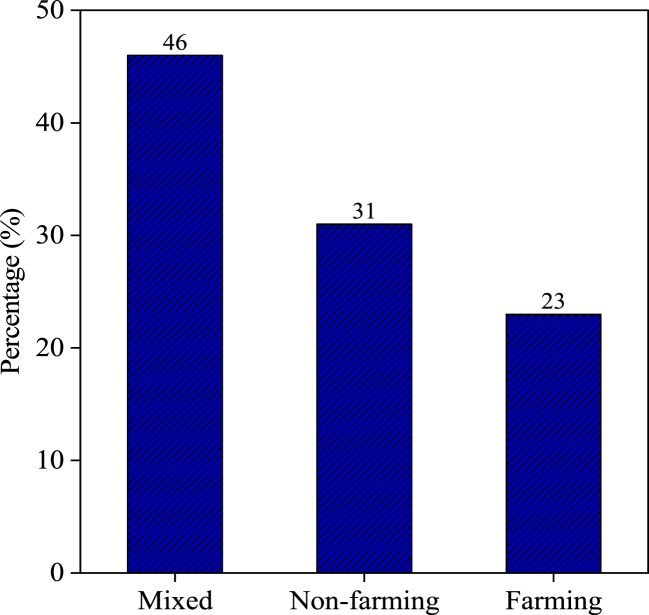
Livelihood strategies of displacees.

In our research, we have identified three primary livelihood strategies among displaced households based on the criteria area recommended by Nielson et al. [93] and Khatiwada et al. [81]. Of these households in Fig. 5, 84 (46 %) derived their income from a combination of farming and employment. In addition, 43 households (23 %) solely relied on farming as their source of income, whereas 58 households (31 %) generated their income through nonfarming strategies.

##### 4.1.3.4 Displacees’ reasons for staying at home

**Fig. 6:**
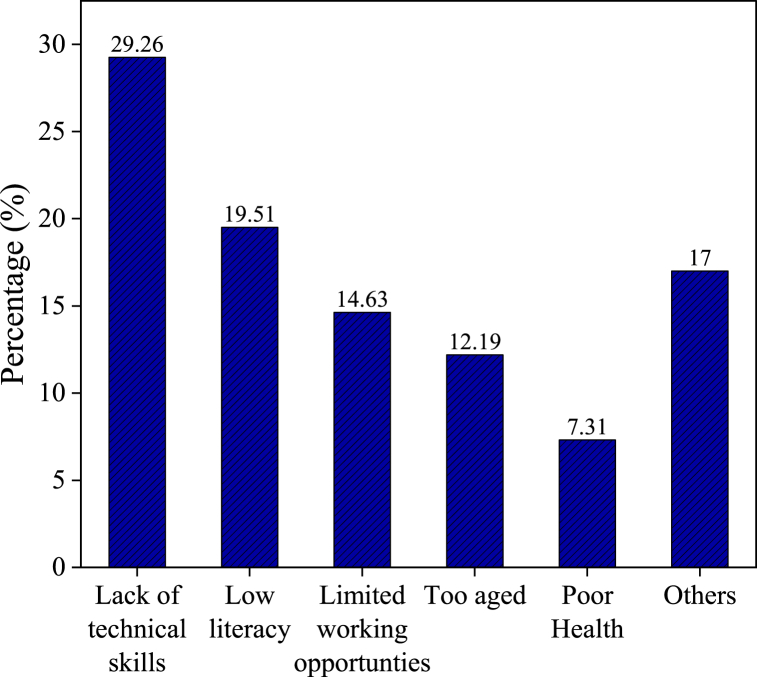
Reasons for staying at home.

In our survey of 185 households, we found that 41 household heads were not engaged in any form of employment activities. In our questionnaire, we had asked the respondents about their reasons for staying at home. Fig. 6 presents the key factors contributing to household heads staying at home. The primary factor was a “lack of technical skills,” mentioned by 29.26 %. “Low literacy” was the second most common reason, cited by 19.51 %, followed by “limited employment opportunities,” reported by 14.63 %. Furthermore, age-related issues affected 12.19 % of the unemployed, whereas “poor health” was a factor for 7.31 %. Other factors mainly included seeking foreign employment, not suitable jobs, waiting for foreign visas, etc.

##### 4.1.3.5 Livelihood outcomes

As mentioned in the analytical framework, we measured the livelihood outcomes of displacees based on daily per capita income and two subjective indicators. These livelihood outcome results are explained below.

##### 4.1.3.6 Change in living standard

**Fig. 7:**
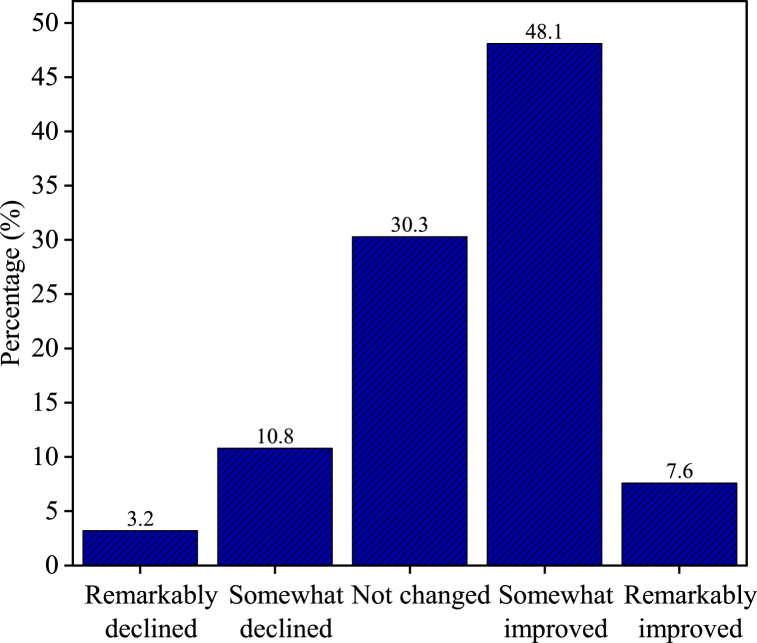
Change in living standard.

Fig. 7 shows data on the changes in the living standards of people displaced by hydropower projects. The findings indicate a diverse impact on the displaced population. A small fraction of respondents (3.2 %) reported a remarkable decline in their living standards, whereas 10.8 % experienced a slight decline. Interestingly, 30.3 % indicated that their living standards remained unchanged. Nearly half of the respondents (48.1 %) observed a slight improvement in their living standards, whereas 7.6 % reported a remarkable improvement. These results indicated a significant proportion of respondents who experienced stability or improvement in their living conditions.

##### 4.1.3.7 Evaluation on livelihood conditions

Table 7 presents an evaluation of livelihood conditions among displaced individuals. The primary concern raised by the respondents were the high cost of children's tuition (14.6 %), followed by efforts to seek employment abroad (13.5 %) and the challenge of unsteady income (13.0 %). In addition, 11.9 % of the respondents reported that their income was too low to improve their living standards, whereas 11.4 % cited high medical expenses for family members as a significant burden. Repayment of debts was an issue for 10.8 % of the participants, and 9.7 % reported that their income was insufficient to maintain a basic standard of living. Supporting elderly family members was a challenge for 8.6 %, and only 4.3 % of the respondents felt no pressure. Lastly, 2.2 % of the individuals reported having no source of income due to job unavailability. These findings indicate the multifaceted economic pressures faced by displaced people.

**Table 7:** Displacees’ evaluation on livelihood conditions.

Items	Frequency	Percent
Children's tuition too high	27	14.6
Trying to find channels and expenses for abroad employment	25	13.5
Unsteady Income	24	13.0
Income too low to improve living standard	22	11.9
Medical expenses too high for family member with diseases	21	11.4
Repayment of debts	20	10.8
Income too low to maintain basic life	18	9.7
Unable to support the elderly family member	16	8.6
No pressure	8	4.3
No source of income with job unavailability	4	2.2
Total	185	100.0

##### 4.1.3.8 Daily per capita income

Table 8 presents the per capita daily income ranges and adjusted poverty percentages for different livelihood strategies among the displaced population. Individuals engaged in mixed livelihood strategies had the highest earning potential, with a minimum daily income of $0.45 and a maximum of $17.81. Nevertheless, 62.65 % of people using this strategy still fell under the adjusted poverty line. Those who relied solely on nonfarming activities had a slightly lower income range, from $0.39 to $10.68 per day, with a higher adjusted poverty percentage of 66.07 %. Pure farming strategies yielded the lowest income, ranging from $0.34 to $4.11 per day, and had the highest adjusted poverty rate of 72.09 %. These figures indicate that mixed livelihood strategies offer the highest earning potential, yet a significant portion of individuals across all strategies remained below the poverty threshold.

**Table 8:** Displacees’ daily per capita income.

Livelihood strategies	Min per capita daily income	Max per capita daily income	Adjusted percentage under poverty (%)
Mixed	0.45	17.81	62.65
Nonfarming	0.39	10.68	66.07
Pure farming	0.34	4.11	72.09

### 4.2 Regression analysis

#### 4.2.1 Variable standardization

Before analyzing various relationships, data normalization is a crucial preprocessing step that ensures the comparability of variables with different scales [98]. To achieve this, we employed the Z-score normalization method. This method involves subtracting the mean of each variable from its values and then dividing the result by the standard deviation. Mathematically, for a variable X_i_ in a dataset D, the Z-score transformation is calculated as shown in Equation (2):

(2) $$Z_{i}=\frac{X_{i}-\mu_{i}}{\sigma_{i}}$$

where:

$Z_{i}$ denotes the Z-score value of $X_{i}$.

$\mu_{i}$ denotes the mean variable $X_{i}$ in the dataset D.

$\sigma_{i}$ denotes the standard deviation variable of $X_{i}$ in the dataset D.

#### 4.2.2 Assumption testing

##### 4.2.2.1 Independence of observations

To check for the independence of our data, we applied the Durbin–Watson test. The Durbin–Watson test measures autocorrelation (also known as serial correlation) in the residuals from a regression analysis. Autocorrelation refers to the similarity of a time series across successive time intervals. It can lead to underestimates of the standard error, potentially causing predictors to appear significant even when they are not.

In our analysis, the Durbin–Watson statistic was calculated to evaluate the presence of autocorrelation in the residuals of the regression model. The results presented in Table 9 show the value of 1.809, which is close to 2. This suggests that there is no significant autocorrelation in the residuals, thus satisfying the assumption of independence of observations required for a valid regression analysis [105].

**Table 9:** Model summary and Durbin–Watson test results.

Model	R	R square	Adjusted R square	Std. error of the estimate	Durbin–Watson
1	0.285	0.081	0.023	0.72789	***1.809***

#### 4.2.3 Principal component analysis (PCA)

PCA is a multivariate statistical method that transforms a large set of correlated variables into a new set of uncorrelated variables known as principal components (PCs). The first few PCs retain the maximum variation present in the original variables [106]. PCA is extensively used in exploratory data analysis owing to its ability to simplify complex datasets. For instance, Jollands et al. employed PCA to evaluate combined eco-efficiency measurements [107], whereas Muzamhindo employed it as a comprehensive ranking method to evaluate university quality [108]. In addition, PCA has been used to determine the sustainability status of manufacturing companies [109], evaluate health infrastructure in Haryana [110], and measure SL in the Indian Sundarban [111].

PCA for livelihood asset indicators simplifies analysis, identifies patterns, reduces noise, handles multicollinearity, and aids in data interpretation and visualization. Hence, PCA was employed in this study.

##### 4.2.3.1 Assumptions and adequacy tests for PCA

The Kaiser–Meyer–Olkin (KMO) measure of sampling adequacy was calculated to determine the suitability of the dataset for PCA. The KMO value was found to be 0.642, indicating a moderate level of sampling adequacy [105]. In addition, Bartlett's test of sphericity was conducted to determine whether the correlation matrix was significantly different from an identity matrix. This test checks if the variables are related and suitable for structure detection. The test results indicated a statistically significant departure from sphericity (x^2^ (55) = 640.321, *p* < 0.05). This suggests that the assumption of sphericity was violated, thereby supporting the appropriateness of PCA for the data.

##### 4.2.3.2 PCA results

**Fig. 8:**
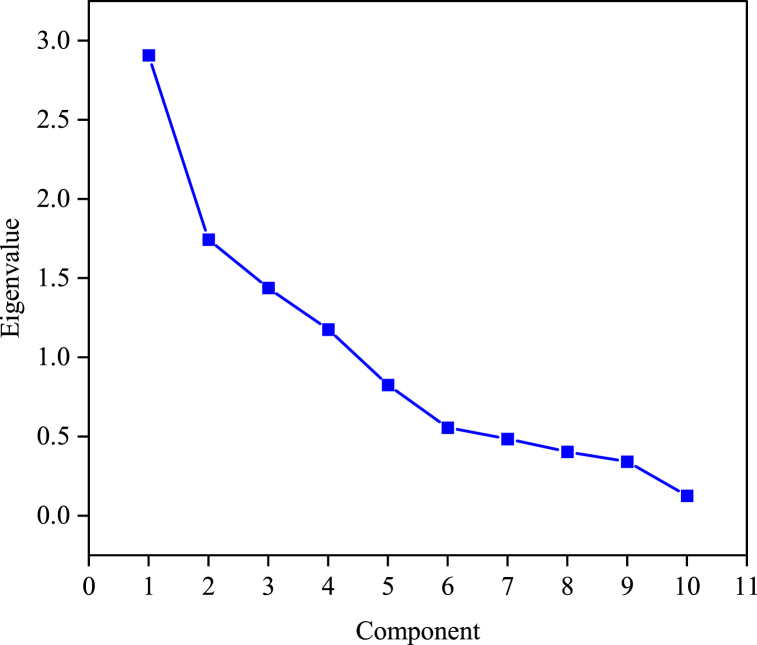
Scree plot of the principal component analysis.

Using the Kaiser criterion for factor extraction, which retains factors with eigenvalues greater than one, four components were determined as shown in Table 10. Together, these four components collectively accounted for 72.673 % of the total variance. To more directly present the PCA results, this study incorporates the factor loading matrix to list the indices with correlation coefficients greater than 0.5. The scree plot of the principal component analysis is shown in Fig. 8.

**Table 10:** Eigenvalues and variance explained by principal components.

Component	Initial eigenvalues	% of variance	Cumulative %
1	2.908	29.076	29.076
2	1.744	17.441	46.518
3	1.439	14.385	60.903
4	1.177	11.77	72.673

The pattern matrix was used to facilitate the interpretation of the PCs. The rotation method applied was Oblimin with Kaiser normalization as per Zhang et al.’s recommendation [29], which converged in 5 iterations. The factor loadings of the variables on the PCs are shown in Table 11.

**Table 11:** Pattern matrix.

Items	HA	PA	SA	FA
Insurance	0.875			
Labor force education	0.860			
Migrant labor	0.803			
Household assets		0.959		
Agriculture tool		0.956		
Contact with relatives abroad			0.832	
Degree of immigration policy perfection			0.820	
Difficulty in democratic decision-making			0.807	
Savings				0.770
Loan source				0.764

Note: Rotation method – Oblimin with Kaiser normalization; PA: physical assets; SA: social assets; HA: human assets; FA: financial assets.

#### 4.2.4 Multinomial regression analysis

A multinomial logistic regression was conducted to predict the livelihood strategies of hydropower displacees, with farming as the reference category. The model fitting information and likelihood ratio tests are shown in Table 12, and the parameter estimates are presented in Table 13.

**Table 12:** Model fitting information.

Measure	Value	Chi square	df	Sig.
*Model fitting criteria*				
Intercept only	392.68			
Final	372.93	19.75	10	0.032
*Goodness of fit*				
Pearson		373.712	358	0.273
Deviance		372.93	358	0.283
*Pseudo R-square*				
Cox and Snell	0.101			
Nagelkerke	0.115			
McFadden	0.050

**Table 13:** Parameter estimates for livelihood strategies.

Livelihood strategies		B	Std. error	Wald	Sig.	Exp(B)	95 % Confidence interval for Exp(B)	
							Lower bound	Upper bound
Mixed	Intercept	0.795	0.212	14.086	0.000			
	HA	0.113	0.197	0.328	0.567	1.120	0.761	1.648
	PA	0.424	0.207	4.188	0.041*	1.529	1.018	2.295
	SA	−0.499	0.229	4.724	0.030*	0.607	0.387	0.952
	FA	0.477	0.277	2.968	0.085^†^	1.611	0.936	2.772
	NA	0.046	0.188	0.059	0.808	1.047	0.724	1.514
Employment	Intercept	0.430	0.225	3.665	0.056^†^			
	HA	−0.100	0.210	0.228	0.633	0.905	0.600	1.365
	PA	0.395	0.219	3.260	0.071^†^	1.485	0.967	2.281
	SA	−0.352	0.239	2.171	0.141	0.703	0.440	1.123
	FA	0.161	0.307	0.274	0.601	1.174	0.644	2.141
	NA	−0.139	0.209	0.443	0.506	0.870	0.577	1.311

Note: The reference category is Farming. Significance levels: *p < 0.05, ^†^*p* < 0.10.

##### 4.2.4.1 Model fit and goodness of fit

The multinomial logistic regression model was statistically significant (x^2^ (10) = 19.750, *p* = 0.032), indicating that the predictors were reliably distinguished between the livelihood strategies of hydropower displacees. The goodness-of-fit tests (Pearson x^2^ (358) = 373.712, *p* = 0.273; deviance x^2^ (358) = 372.930, *p* = 0.283) suggest that the model fits the data well. The pseudo-R-squarevalues indicate that the model explains a modest portion of the variance in livelihood strategies (Cox and Snell = 0.101, Nagelkerke = 0.115, McFadden = 0.050).

##### 4.2.4.2 Parameter estimates for livelihood strategies

Table 13 shows the multinomial regression parameter estimates for the different livelihood strategies compared with the reference category of farming. For the mixed livelihood strategies model, the intercept was significant (B = 0.795, SE = 0.212, *p* < 0.001). Among the predictors, PA had a significant positive effect (B = 0.424, SE = 0.207, *p* = 0.041) on mixed livelihood strategy, suggesting that an increase in physical assets increases the odds of adopting a mixed livelihood strategy (odds ratio: 1.529, 95 % confidence interval, [1.018, 2.298]). SA had a significant negative effect (B = −0.499, SE = 0.229, *p* = 0.030) on mixed livelihood strategy, suggesting that an increase in social assets decreases the odds of adopting a mixed livelihood strategy (odds ratio: 0.607, 95 % confidence interval: [0.387, 0.952]). FA also showed a marginally significant positive effect (B = 0.477, SE = 0.277, *p* = 0.085) (odds ratio = 1.611, 95 % confidence interval = [0.936, 2.773]). However, HA and NA were not found to be significant predictors in this model.

In the employment strategy model, the intercept was marginally significant (B = 0.43, SE = 0.225, *p* = 0.056). PA showed a marginally significant positive effect (B = 0.395, SE = 0.219, *p* = 0.071) (odds ratio = 1.485, 95 % confidence interval = 0.967, 2.281), indicating that an increase in physical assets might increase the odds of adopting an employment strategy. However, HA, SA, FA, and NA were not found to be significant predictors in this model.

In summary, the mixed livelihood strategies model showed that physical and social assets are significant predictors, with physical assets increasing and social assets decreasing the likelihood of adopting mixed livelihood strategies. The employment strategy model indicated a marginally significant effect for physical assets, indicating a potential increase in the likelihood of adopting employment strategies with increased physical assets.

## 5 Discussion

Our findings provide insight into the complicated dynamics of livelihood strategies among households displaced by the Tanahu Hydropower Project. We identified major elements influencing the decision-making processes of Tanahu hydropower displacees by evaluating the impact of various livelihood assets. The findings highlight the importance of physical assets in facilitating livelihood diversification as well as the limitations associated with reliance on agriculture-based livelihoods. Overall, our findings indicate a poor relationship between livelihood assets and strategies. This discussion connects our findings with current research, providing a detailed view of the difficulties impacting displaced populations and policy solutions to facilitate their long-term rehabilitation and resilience.

### 5.1 Influence of livelihood assets on strategy choices

The multinomial logistic regression analysis revealed significant insights into the impact of different livelihood assets on the choice of livelihood strategies. For the mixed livelihood strategies model, the significant intercept indicates a strong baseline propensity for households to adopt diversified income strategies. Among the predictors, PA was found to have a significant positive effect, suggesting that households with greater access to physical resources, such as infrastructure and equipment, are more likely to diversify their income sources. This finding is consistent with those of previous studies that emphasized the importance of physical capital in enhancing economic resilience and promoting diversified livelihood activities [52,81,88].

Social assets refer to social resources people utilize to achieve their life goals. They can be used to provide the assistance the impoverished require and, to some extent, lower the cost of accessing resources with protection [88,98]. As an external factor, SA is crucial in raising people's living standards [52]. However, SA had a significant negative effect on the likelihood of adopting mixed livelihood strategies. This unexpected finding indicates that the pursuit of a variety of revenue-generating activities may occasionally be hampered by strong community ties and support networks, which are often viewed as beneficial.

Furthermore, financial assets (FAs) showed a marginally significant positive effect, indicating that households with better financial resources are somewhat more likely to engage in mixed livelihood strategies. This is consistent with other research, which demonstrated that having additional funds helps families think of other sources of income [81,112]. However, the influence of FAs was not as strong as that of physical assets.

However, HA and NA were not significant predictors in the mixed-livelihood strategy and employment model. This lack of significance may be due to the relatively low average level of education among the households surveyed. With an average educational level just above primary school, the impact of human capital on livelihood strategy choices might be limited. In addition, this insignificance can be attributed to the fact that subsistence farming alone cannot sustain the livelihoods of rural households with smaller land sizes [81]. These households are compelled to generate higher income from their limited land through commercially oriented farming and nonfarming activities. Thus, merely owning land does not significantly influence the likelihood of adopting mixed or employment-based livelihood strategies.

In the employment strategy model, the intercept was marginally significant, indicating a baseline tendency for households to consider employment-based strategies. Physical assets again exhibited a marginally significant positive effect, highlighting the importance of physical capital in enabling households to pursue employment opportunities. Households with better physical resources may have better access to transportation, communication and other infrastructure necessary for securing and maintaining employment [88]. Moreover, the overall research finding is consistent with that of Diaz et al., which highlighted the fact that in the case of dam construction, the physical assets have a positive impact. Simultaneously, other assets such as natural, human, and financial are negatively impacted in the global south. This is because dam construction needs better infrastructure, such as roads, to successfully conduct their operations [2].

### 5.2 Livelihood aspirations and compensation satisfaction

Our study of the livelihood goals of displaced people revealed a wide range of goals, from the basic desire for improved living conditions to the generational emphasis on children's development. One of the main pillars that emerged was financial security, highlighting the importance of economic stability in determining aspirations for a living. These hopes reflect the complex objectives of displaced households, which include both short-term financial demands and long-term aspirations for progress.

The Tanahu Hydropower Project's compensation schemes yielded varying degrees of satisfaction among the respondents. However, some expressed their happiness with the remuneration for the improved living conditions and appropriate policies, as many of the households were able to use compensation money to build new houses, pay for children's education, and start small businesses. However, some were reluctant to give up their land and were particularly unhappy. The issues around perceived lower compensation rates in comparison to other places, higher living costs, and increasing everyday expenses all point to serious difficulties in heavily relying on the cash compensation process. These results are consistent with previous research emphasizing the value of strong policy frameworks and support networks for effective relocation and livelihood rehabilitation [94,113].

### 5.3 Livelihood strategies and poverty

The study identified three primary livelihood strategies among displaced households: mixed strategies, pure farming, and nonfarming. The high prevalence of mixed strategies indicates that households are attempting to mitigate income instability by diversifying their sources of livelihood. However, the substantial proportion of households solely relying on farming and the associated high poverty rates signifies the vulnerability of agriculture-based livelihoods, particularly in displacement settings.

The primary reasons for staying at home included a lack of technical skills, low literacy, and limited employment opportunities. These barriers to employment indicate the importance of targeted skill development initiatives to enhance employability and economic self-sufficiency among displaced individuals.

### 5.4 Livelihood outcomes and economic pressures

The analysis of livelihood outcomes based on daily per capita income and subjective indicators yielded mixed results. While a significant proportion of respondents reported stability or improvement in their living standards, a substantial fraction experienced a decline. The high cost of children's tuition, efforts to seek employment abroad, and unsteady income were major concerns affecting displaced households. These economic pressures indicate the varied challenges faced by displaced individuals and the need for comprehensive support systems to address these issues.

Daily per capita income analysis revealed that mixed livelihood strategies exhibited the highest earning potential but also had a substantially adjusted poverty rate. Nonfarming and pure farming strategies yielded lower incomes and higher poverty rates, highlighting the need for policies that promote income diversification and economic resilience. The findings align with those of previous research on the economic vulnerabilities of displaced populations and the importance of diversified livelihood strategies for sustainable recovery [79].

### 5.5 Policy implications and recommendations

Our results indicate how crucial it is to implement targeted policy interventions to assist displaced households in reestablishing their standard of living. It is important to implement efficient rules for asset management and compensation to facilitate the effective transition of displaced households to new sources of income. Long-term economic stability can be promoted by investments in physical infrastructure, such as improved road connectivity, which can increase access to opportunities for work and education. Similarly, considering the negative impact of social assets on livelihood decisions, policy interventions should not solely focus on building social capital. Instead, they should acknowledge the limitations of close community ties, foster individual economic choices, and promote diverse social networks to balance social and economic goals.

In addition, to meet the present and future financial demands, financial support mechanisms such as long-term microcredit programs and emergency cash assistance are imperative. Programs for skill development and vocational training must be given top priority if displaced people are to become more employable and financially independent. Finally, increasing land productivity and offering subsidized agricultural inputs are key to mitigating the insignificant influence of natural capital. Addressing the issues of low productivity and farmland abandonment can support long-term agricultural livelihoods.

### 5.6 Limitations of the research

Our study employed an asset-based approach and mainly relied on questionnaires for data collection. It is important to note that the nature of assets can evolve, particularly in situations involving displacement. The perception of what constitutes an asset can shift as people adapt to their new surroundings post displacement. Similarly, the sample size was limited to 185 households, which, although accounting for 30 % of the displaced population, may not capture the full diversity and experiences of all displacees. Moreover, some residents who received large sums of cash compensation have already migrated to different parts of the country. Hence, future research with longitudinal studies and findings of those who moved away from the original settlement and comparing them with those who stayed would give a new insight into the livelihood situation of displacees.

## 6 Conclusion

The paths taken by displaced populations in terms of livelihood are complex and diverse. Our investigation into the goals, strategies, and difficulties faced by these communities revealed a complicated web of variables influencing their life after displacement. Their assets are essential to these aspects and have a significant impact on strategic decisions. Although having sufficient finances is still the major objective, having access to physical resources, such as marketplaces, roads, and machinery, is crucial in influencing people's choices. Notably, financial and human capitals are equally important for resilience and recovery; however, our data showed that they had little effect on livelihood strategy decisions. Supporting sustained resettlement and rehabilitation requires an understanding of the many components, which include short-term financial assistance and long-term skill development initiatives. The low level of economic stability in the areas under study is highlighted by the majority of the population's daily income being below the international poverty line. Hence, it is important to address the complex challenges faced by hydropower displacees. A carefully designed benefit-sharing plan that fully satisfies their requirements is essential. Such a plan aims to make impacted communities active participants in the development process, guaranteeing their long-term benefits instead of just cash compensation.

## Ethics approval and consent to participate

This research was approved by the China Three Gorges University Institutional Review Board (IRB2021KF001). Verbal consent was obtained from all subjects during the survey.

## Funding

This work was supported by the 10.13039/501100012325National Social Science Fund of China (Fund No. 21 & ZD 183), Community Governance and Post-relocation Supporting on Cross District Resettlement, and the fund of Research Center for Reservoir Resettlement, China 10.13039/501100002861Three Gorges University (Grant No. 2021KFJJ01).

## Ethical issues

There are no ethical issues.

## Data availability

Data for this research is available at Mendeley Data, https://doi.org/10.17632/cbvxkkgy38.3 after the embargo ends.

## CRediT authorship contribution statement

**Ribesh Khanal:** Writing – review & editing, Writing – original draft, Methodology, Formal analysis, Data curation. **Yuefang Duan:** Writing – review & editing, Validation, Supervision, Funding acquisition, Conceptualization. **Thomas Stephen Ramsey:** Writing – review & editing. **Sher Ali:** Writing – review & editing. **Kaung Htet Oo:** Writing – review & editing.

## Declaration of competing interest

The authors declare that they have no known competing financial interests or personal relationships that could have appeared to influence the work reported in this paper.
